# Attitudes and doping: a structural equation analysis of the relationship between athletes' attitudes, sport orientation and doping behaviour

**DOI:** 10.1186/1747-597X-2-34

**Published:** 2007-11-09

**Authors:** Andrea Petróczi

**Affiliations:** 1 Kingston University, School of Life Sciences, Penrhyn Road, Kingston upon Thames, Surrey, UK, KT1 2EE

## Abstract

**Background:**

For effective deterrence methods, individual, systemic and situational factors that make an athlete or athlete group more susceptible to doping than others should be fully investigated. Traditional behavioural models assume that the behaviour in question is the ultimate end. However, growing evidence suggests that in doping situations, the doping behaviour is not the end but a means to an end, which is gaining competitive advantage. Therefore, models of doping should include and anti-doping policies should consider attitudes or orientations toward the specific target end, in addition to the attitude toward the 'tool' itself.

**Objectives:**

The aim of this study was to empirically test doping related dispositions and attitudes of competitive athletes with the view of informing anti-doping policy developments and deterrence methods. To this end, the paper focused on the individual element of the drug availability – athlete's personality – situation triangle.

**Methods:**

Data were collected by questionnaires containing a battery of psychological tests among competitive US male college athletes (n = 199). Outcome measures included sport orientation (win and goal orientation and competitiveness), doping attitude, beliefs and self-reported past or current use of doping. A structural equation model was developed based on the strength of relationships between these outcome measures.

**Results:**

Whilst the doping model showed satisfactory fit, the results suggested that athletes' win and goal orientation and competitiveness do not play a statistically significant role in doping behaviour, but win orientation has an effect on doping attitude. The SEM analysis provided empirical evidence that sport orientation and doping behaviour is not directly related.

**Conclusion:**

The considerable proportion of doping behaviour unexplained by the model suggests that other factors play an influential role in athletes' decisions regarding prohibited methods. Future research, followed by policy development, should incorporate these factors to capture the complexity of the doping phenomenon and to identify points for effective anti-doping interventions. Sport governing bodies and anti-doping organisations need to recognise that using performance enhancements may be more of a rational, outcome optimizing behaviour than deviance and consider offering acceptable alternative performance-enhancing methods to doping.

## Background

The fight against doping is a challenging task. Owing to the complexity of the doping phenomenon, simultaneous consideration of physiological, medical, pharmacological, psychological, ethical and systemic factors [1] is required in order to be successful in this endeavour. The need for effective deterrence policy is underscored by the fact that the problem of performance enhancements has spread beyond the elite athlete population. It is well documented that groups other than competitive athletes are at risk of using doping agents, especially steroids [2-5]. Furthermore, medical enhancement of non-sport performance (i.e. quality of life, appearance) appears to be widely acceptable among non-athlete population [6-8].

For this this paper, the term 'doping' is used as the employment of prohibited means to enhance performance, with the intention to gain competitive advantage over the opponent. This definition incorporates the key elements of the previously used definitions, such as artificial stimulation [9] and intention to gain advantage [10], and it is also congruent with the current official definition [11]. Doping may be done by taking prohibited (banned) performance enhancing drugs (PEDs) or using banned methods. Using agents to mask the presence of PEDs, physical manipulation and tampering with the test samples, non-therapeutic gene manipulation resulting in enhanced sport performance and non-cooperation of an athlete are also considered doping. The list of prohibited substances is published by the World Anti-Doping Agency (WADA) and updated yearly [11]. The WADA operates a Therapeutic Use Exemption scheme to allow athletes to take prohibited substances if they have an illness or medical condition that requires medication otherwise on the Prohibited List. Using supplements that are not on the Prohibited List does not constitute doping even if they have a performance enhancing effect (e.g. caffeine, creatine, protein).

Recreational drugs (also called social drugs) are psychoactive drugs used for recreational purposes rather than for work, medical or spiritual purpose, although some recreational drugs (e.g. marijuana, hasish, heroin, amphetamine, ephedrine) are on the List of Prohibited Substances if they the concentration in urine exceeds a specified level and/or were taken during competition.

### The doping phenomenon

Despite the fact that doping is not a new phenomenon in sport, enhancing performance through artificial means has only been banned since the 1960s. Doping as a potential danger to the modern Olympic movement was recognized in the '50s and officially acknowledged ten years later by the creation of a list of banned substances. After an agonizing period over athletes' amateur status, performance enhancing drugs have taken over as the major basis for tension and concern within the Olympic movement since 1972 [12]. Researchers seem to agree that doping is unwelcome in sport. However, opinions are divided between doping being a serious deviance one must fight against and doping as undesirable but unavoidable consequence of the institutionalized sport. Notably, the reason behind banning doping initially was the growing concern about athletes' health [13]. Doping only became established as unethical after that point.

Doping incidents infesting high prestige sport events such as the 1998 Tour de France, which was dubbed as the 'Tour of Shame' [14] or the 2004 Athens Olympic Games with a sudden double number of positive cases [15]; and the reaction to them (i.e. establishing national anti-doping agencies) indicate that these events may only be the tip of the iceberg. Whilst the adverse analytical findings (positive results) in tests conducted by the World Anti Doping Agency (WADA) remain low around 2% [16], other occassions have revealed an elevated level of substance use. For example, the presence of some kind of drug or supplement was evidenced in 45% of the athletes who participated and were tested in the Tour de France 2000 [17]. However, the problem seems to be rooted more deeply. The litereature supports the assumption that the consideration of and actual use of doping starts well before the athlete reaches his/her best career years as the prevalence of doping, particularly the use of anabolic steroids, is well documented among adolescents [18-21] and even among pre-adolescent athletes where a steady increase in doping use was observed over the period of four years from age 11 to 15 [22].

The seriousness of the problem is reflected by the recent increase in organised effort to combat doping in sport. The first step toward a globalised effort was the creation of the Anti-Doping Code of the World Anti-Doping Agency (WADA) in 1999 as an organisational level response to the Festina Scandal at the Tour de France [14], parallel to the European Union's (EU) pledged support in the fight against doping. The first report (known as the HARDOP report) was commissioned in 1998 and published in 1999, followed by targeted research projects under the EU's Competitive and Sustainable Growth run under 5^th ^Framework Programme [23]. The globalised effort was recently manifested in the creation of the International Convention Against Doping in Sport by the United Nations Educational, Scientific and Cultural Organization (UNESCO) [24]. The UNESCO convention is the first legally binding international framework setting out the responsibilities of national governments and is currently signed either as ratification, acceptance, approval or accession by 65 countries.

### Doping prevention

Historically, the anti-doping movement has been based on detection and prevention, with the initial emphasis on detection. Organisational structures and standard operating procedures have been in place to ensure compliance with the anti-doping regulations [25]. Detection relies on testing, which has been increasingly problematic in high performance sport. Haugen [26] argued persuasively that making testing effective as a deterrence method, either the volume of tests conducted or the sanctions imposed have to be increased significantly, potentially to the level that is practically not feasible. The new technologies in both the development of undetectable methods and the detection of the new methods have led to rapidly escalating costs [27], bearing in mind that tests are currently not even available for all banned substances and methods. If the trend continues, costs of effective testing will soon became a prohibiting factor.

Athletes, as they progess in their sports career, are gradually drawn into the vicious circle of the constant desire to enhance performance. In this process, some athletes may become more susceptible to doping than others, depending on the combination of their personality and the situation. Therefore, both the individual and systemic factors contributing to doping behaviour should be fully investigated in order to underpin effective, targeted anti-doping intervention.

In support of the argument against detection from a psychological perspective, Strelan and Boeckmann [28] provided empirical evidence for the failure of detection based deterrence showing that in a hypothetical situation, athletes first consider their moral beliefs, followed by the fear of negative health consequences and legal sanctions associated with the use performance-enhancing drugs. The effect of the threat of legal sanctions practically diminished when moral beliefs and health concerns were added to the behavioural model, directing policy makers to alternative deterrence methods. Additionally, many speculate that with gene doping on the horizon of competitve sport, detection based regulation will soon be seriously undermined [29-33].

The WADA and national sport governing bodies have added preventive measures to their detection programs. Examples for anti-doping prevention include: WADA's *Athlete Outreach Program *(launched in 2001) targeting top performing athletes at major sporting events, the *Anti-Doping Development Program *(started in 2004), which aims to help countries and organizations to set up quality doping control, and the *Educational Programme*, which is a major tool of the WADA in an attempt to create a doping free culture by providing education to all stakeholders about the dangers of doping and its consequences.

Congruently, the *100% me *programme of UK Sport aims to promote positive attitudes and values of those who successfully competed drug-free and to provide accurate and relevant information on anti-doping. The *100% me *is an educational program with three distinct but related strands. Outreach programme provides a framework for delivering accurate information and giving advice on anti-doping issues to athletes, athlete support personnel, and parents across the UK via sports events, workshops, training sessions and conferences. The accreditation programme allows interested individuals to gain knowledge in anti-doping and became a '*100% me' *tutor. The *100% me *is also a 'brand' promoting the image of the 'clean athletes' based on values of personal responsibility, choices, fairness and honesty. This image is linked to the Ambassador programme where successful drug-free athletes committed to anti-doping use the *100% me *platform to promote drug-free sport among their fellow athlete The *Education Model Guidelines *(EMG) are in place to help National Governing Bodies (NGBs) develop their own programmes using the *100% me *framework.

The UK model is one of the existing anti-doping national programmes. In the US, the U.S. Anti-Doping Agency (USADA) is responsible for similar testing and education programmes, and in place to eliminate conflict of interest of NGBs testing and sanctioning their own athletes. The Australian Sports Anti Doping Authority (ASADA) has also launched a comprehensive the *ASADA Education Service Charter *in 2007. The Charter places an emphasis on developing athletes' and support personnels' understanding of the physical and psychological risks of doping to ensure that athletes and support personnel are aware of their rights and responsibilities.

Despite the increased anti-doping effort, the relative number of adverse analytical findings has not decreased considerably in the past four years [16]. The appropriateness of education as a deterrent is questionable as it has been shown that doping specific knowledge is higher among doping users than among their non-user counterparts [34]. While prevention, complemented with detection, will be likely to be the main approach to the doping problem, the ultimate goal for sport governing bodies should be creating policies for a truly effective deterrence. Setting detection aside, there is still a fundamental distinction between prevention and deterrence. It is suggested that prevention (and detection) create an environment where the chances of detection and punishment for using doping are uncomfortably high, hence keep athletes away from employing such means, regardless of their motives. On the other hand, value-based deterrence in its true, perhaps Utopian sense, is associated with the creation of an environment where athletes never feel motivated to use illegal means for performance enhancement.

Whether it is a realistic goal or not, effective deterrence is hindered as long as doping behaviour is poorly understood. Before any serious consideration is given to deterrence methods, factors that make an athlete or athlete group more inclined to doping than others must be fully investigated. The WADA has only just started to channel funds to social science doping research to develop better understanding and consequently, more effective deterrence programs. Aiming to add to the body of knowledge on one possible cause of doping behaviour (i.e. individual dispositions and attitudes) is congruent with the current priorities of the WADA Social Science Research Programme [35].

### Explaining the doping behaviour

Both the eminent literature and the official global sport organisational stance suggest that athletes' attitudes are responsible for the deviant behaviour of doping [36-38]. Being overly competitive or exceedingly win-orientated is often used as a lay explanation for doping. Although gender, cultural and competitive level differences among athletes have been scrutinized since the late '80s [39,40] the relationship between these factors and doping behaviour has not been empirically tested, except in one project. In the study by Lucidi et al. [41] the classic *Theory of Planned Behaviour *(TPB) model [42] provided a theoretical framework for a study among Italian adolescents, where attitude was found to be the strongest predictor for behavioural intention. The TPB model held across different levels of sport involvement and gender.

Recently, alternative theoretical models of doping have been developed [43,44] attempting to explain the complex nature of doping. The models are based on existing general models from either health science or criminology but their application to the doping situation has not been empirically validated. The first among the few, Donovan and colleagues [43] used the *Health Belief Model *to develop a theoretical drug control model. Although it was not explicitly stated, the model also incorporates some kind of economic rationality when it considers the balance between deterrence and incentives and availability and affordability of performance enhancing substances. According to the model, athletes' doping behaviour is the ultimate function of this cost/benefit ratio, personality and morality, legitimacy of sanctioning organisation, social context (reference group) and attitude toward doping.

The *Drugs in Sport Deterrence Model *by Strelan and Boeckmann [44] also considered costs and benefits but used these concepts in a broader sense. Their model is based on *Deterrence Theory *used in criminology [45] and costs and benefits include material and social consequences, as well as individual effects, such as health concerns, guilt or even satisfaction from sport achievement. Situational factors (i.e. prevalence perception, professional status, type of drug, experience with testing, etc.) were also thought to have an effect on the final decision regarding doping use.

The common element of all three models [41,43,44] is that subjective norms play a seemingly important role in doping behaviour. As it is evidenced in a recent, WADA Social Science research funded extensive literature review [46], published research into doping attitude is dominantly descriptive and with a few exceptions, it falls short on theoretical underpinning or on establishing causal relationships between attitudes and behaviour. The major achievement of the existing doping models is that they draw attention to the complexity of the doping problem. Many of them touched upon attitudes and many other perhaps important factors contribution to doping but their claims have not been supported with empirical evidence.

Therefore, the intention of this study was to to fill this gap and to explore the relationship between doping behaviour and sport achievement orientation by expanding the traditional one-step attitude – behaviour models (e.g. *Theory of Reasoned Action, Theory of Planned Behaviour*) and collecting and analyzing data regarding athletes' sport achievement orientation, doping orientation and behaviour. The traditional one-step behavioural models [42,47] assume that the behaviour in question is the ultimate end and considers antecedents, such as beliefs, attitudes, subjective norms and perceived behavioural control regarding the particular behaviour. Research into athletes' motivation and reasons for doping use reveal an important factor that has been prominent in game theory models [26] but overlooked in the existing doping behaviour models [43,44]: that doping behaviour is not the ultimate end but rather a means to an end [10,48,49]. It can be argued whether the ultimate end is winning or achieving a specific sport related goal (i.e. breaking a record); and it may vary from athlete to athlete. Nevertheless, if doping is a tool to achieve an end-goal, then models of doping should include attitudes or orientations toward the specific target end, in addition to attitudes toward the 'tool' itself.

## Aims

The aims of the study were to investigate athlete's orientation toward doping and estimate the extent to which athletes' personal traits (e.g. competitiveness, win and goal orientation [50] and doping orientation (doping attitude and beliefs) are related to doping behaviours. To this end, this paper focused on the individual element of the drug availability – athlete's personality – situation triangle.

In consideration of the structural model of doping, the existing literature, more specifically the *Theory of Reasoned Action (TRA) *[47], the *Theory of Planned Behaviour (TPB) *[42], previous structural equation models of attitude and behaviour [51-53] and previous doping models [40,42,43,54,55] were consulted. The *Theory of Reasoned Action *[47] established a linear sequence of cognition (beliefs), affects (attitude), conation (behavioural intention) and behaviour. Later, the model has been criticized for the underlying and unrealistic assumption of absolute behavioural control, hence perceived behavioural control was added and the model expanded into the TPB [42]. Models have been empirically tested and refined by showing interaction between the predictors [53] and by questioning the generality of the model [52]. An earlier model of Bentler & Speckart [51] suggests an important notion, namely multiple factors influencing the behaviour. The notion of multiple factors is, of course, not new. In 1977, Ajzen and Fishbein [56] already mentioned multiple-act criterion, where attitudes toward a target (i.e. doping in general) is linked with observed heterogeneous behaviours (i.e. supporting the anti-doping movement but using doping at the same time). Influencing factors can be learned experiences (past behaviour), perceived control, personality, cost/benefit ratio and most importantly: goals. Bentler and Speckart [51] focused on past behaviour (in general) whereas new doping models consider personality, availability, free choice of actions [43] and situational factors as well as perceived control over behaviour and free choice [44]. Curiously, a situation where multiple attitudes are influencing a single behaviour has not been considered in doping attitude-behaviour modeling. If doping behaviour is considered as a means to an end, attitude toward the end point should be taken into account. Support for this assumption can be found in the literature for at least 2 decades.

English [48] suggests that doping may be used to achieve one or more of many goals, including reaching unattainable goals, breaking off the plateau, or even to signal group membership; or mark transition from being recreational to professional athlete. Contrary to Lüschen's argument [10] that the crucial element of doping is the intent to gain unfair advantage at the expense of other competitors, athletes do not necessarily see using doping as unfair or advantageous. Doping may be employed as a useful tool to improve performance to the level that is, or perceived to be necessary to have a reasonable chance for winning [49]. When athletes assume that their competitors follow the same logic, the motivation for doping use is often reduced to the desire to level the playing field and ensure equal chances. These rational decisions regarding doping behaviour could easily be against the general attitude toward doping, which is suppressed by other, stronger driving forces such as the desire to win, goal orientation or competitiveness.

Therefore the aim of this paper is to test a model that embrace the two constucts presumed to be related to winning. The present investigation makes an attempt to provide *empirical evidence *regarding the interconnection of sport orientation, doping attitude, and behaviour. It is not the intention to test an existing doping model or to apply a general attitude-behaviour model to doping situation, but to draw attention to the complexity of the influencing factors in doping behaviour by addressing the missing segment and focusing on a popular lay explanation of athletes' doping behaviour by treating doping behaviour as a means to an end (winning), hence considering elements of the *sport achievement orientation *as integral parts of the doping behaviour model. To this end, athletes' sport orientation and attitude toward doping were quantified, measured by scales, and statistically analysed. Sport orientation and doping orientation are thought to be hypothetical constructs, which cannot be observed or measured directly. Thus, using statistical techniques that are able to represent dependency between hypothetical constructs (latent variables) was preferred. From the array of available statistical techniques, structural equation modeling (SEM) was selected with the main advantage being its applicability to multivariate data where measurements are not expected to be error-free.

## Methods

**Figure 1:**
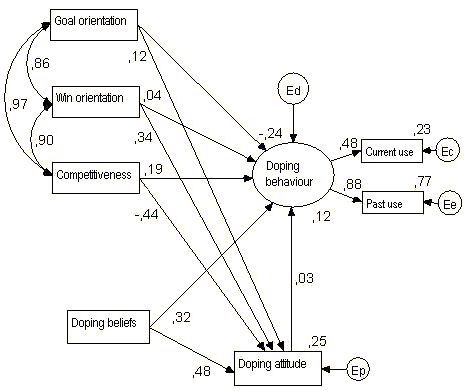
The scale-level model of doping.

In order to investigate the relationship between individual attitudes and behaviour, empirical data were collected via paper and pencil questionnaire from 199 US male college athletes. Athletes' sport orientation and attitude toward doping were quantified and measured using the *Performance Enhancement Attitude Scale (PEAS, see Appendix) *[54] and the *Sport Orientation Questionnaire *[50]. The relationships between use of performance enhancements, attitudes toward performance enhancements, competitiveness, winning, and personal goals were investigated using structural equation modeling (SEM) and the hypothesized models are depicted as a model diagram (Figure 1). Latent variables are presented by ovals with single-headed arrows pointing toward their measured indicators, presented in rectangles. Single-headed arrows from one measured or latent variable to another symbolise effect and two-headed arrows represent correlations. Owing to the discrepancy between the numbers of indicators to their respective latent variables, the hypothesized relationship among attitude and behaviour was tested at item level (not shown) and at measurement scale level (Figure 1). Testing at the item level offers the advantage of examining measurement issues and removing measurement error from the model [57] but requires larger sample size than a single composite indicator structural equation model. Consequently, the model was also tested at the single indicator measurement scale level. The metric for each factor was set by fixing the factor loading for the first item to 1.0.

The proposed models were tested using the maximum likelihood (ML) method in AMOS 5.0. Although the ML method assumes normally distributed data measured on continuous scales, much social science and behavioural research uses ML because of its robustness. As alternative methods are less robust and require very large samples [58], ML methods appeared to be the most suitable for the present study. As a reassurance, the overall model fit statistic was checked against its boostrap equivalent and indicated by the Bollen-Stine *p *value [59]. The model's overall fit was examined using chi-square statistics along with the root mean squared error of approximation (*RMSEA*) and its 90% confidence interval, as well as the standardized root mean square residual (*SRMR) *following Boomsma [60]. As recommended by Hoyle and Panter [61], the Tucker-Lewis index (*TLI*), which is relatively independent of sample size and Bentler's comparative fit index (*CFI*) was also used and reported. All statistical analyses in this study, including SEM, were performed using raw data.

Concerning sample size, there is no clear rule regarding how many subjects are required, yet recommendations [62,63] are fairly consistent about the following: 1) sample sizes under 100 require at least two indicators for every latent variable, 2) sample sizes between 150 and 200 are more desirable, and 3) researchers should try to have at least 5 subjects for every estimable parameter. Thus, a sample size around 150 was planned with two or three indicators for each construct, allowing up to 30 parameters.

### Participants

Because attitude is not thought to be constant across different populations, the sample was drawn from approximately the same population. One hundred and ninety nine male athletes from the US participated in the study. Data from a single-gender sample were collected in order to reduce the within sample variability. Literature precedence suggests that there is a difference in attitude and use of drugs between males and females, males being more likely to endorse using performance enhancing substances [46].

Athletes were approached via their coaches. Participation was voluntary and athletes were assured of complete anonymity. Athletes were informed about the purpose of the study and were made aware that their answers will be used in an aggregated form for research. The survey packets were distributed and collected in individually sealed envelopes by either the athletic directors or coaches in a practice setting. The researchers did not have direct contact with the athletes as the collected surveys were returned to the researchers by mail.

Participants were predominantly from the National Collegiate Athletic Association (NCAA) upper divisions (*n*_DivI _= 78, *n*_DivII _= 116, *n*_DivIII _= 5) and 8 collegiate sports: baseball, basketball, football, golf, ice hockey, soccer, swimming and track and field. The mean age was 20.20 (*SD *= 2.15) and the mean number of years in the sport career was 8.77 (*SD *= 4.68). Information relating to the weekly training hours was not collected. Being a student athlete under the NCAA Bylaw, the allowable training hours are limited to 20 hrs per week during academic season, although anecdotally in some sports (i.e. American football) the 20-hour limit is regularly exceeded. As the sample was recruited from predominantly the upper divisions, it can be assumed that athletes in the sample trained at least 20 hours per week.

The proportion of missing values remained under 10% and appeared to be due to randomly missed items in scales. Cases with missing values were deleted from the data set, resulting in 174 complete cases being used for model fitting. Questions regarding past and present doping behaviour were more problematic with a high proportion (49–50%) of denied answers. Assuming a medium effect size (Cohen's *d *= 0.3 – 0.5), the minimum recommended sample size for most statistical analysis at a 0.05 significance level, and power of 0.7 ranges between 59 and 139 [64-66]. For the purpose of this study, an effect size smaller than 0.3 was considered trivial, thus it was not the researchers' intention to find differences with ES approximately 0.5. Therefore, the final number of participants (*N *= 174) was sufficient (minimum 5 participants per variable, as recommended by Bentler and Chou, cited in Schumacker & Lomax [67]) for calculating internal consistencies, correlation coefficients, comparison, and structural analysis.

### Measures

The survey packet contained the *Performance Enhancement Attitude Scale *(PEAS, [54], the *Sport Orientation Questionnaire *[50], Doping Use Belief (DUB) statements, questions regarding past experience and current use of doping, brief definitions of terminology (i.e. performance enhancing drugs and methods), a cover letter explaining the purpose of the study, and a specially marked envelope for the completed surveys to ensure complete anonymity. To ensure that none of the instruments were seriously confounded by socially desirable responding, the *Social Desirability Scale *[68] was also administered.

The PEAS is a measure of general doping attitudes. Doping attitude is defined as an individual's predisposition toward the use of banned performance enhancing substances and methods. The PEAS consists of 17 attitude statements measured on a six point Likert-type scale ranging from strongly disagree (1) to strongly agree (6). No neutral middle point was offered and all 17 items were scored in the same direction (see Appendix). There was evidence from previous use that the scale is unidimensional and reliable, with Cronbach alpha values above .70 [54,55]. The internal consistency of the scale for the present sample (Cronbach α = .83) was above the customary cutoff value.

*Doping Use Belief *measures (DUB) were operationally defined as expressions of presumed opinion regarding doping use, namely whether doping should be allowed for top and all level athletes (2 separate questions). Participants were asked to select one of the three responses: 'yes, without restrictions', 'yes, with restrictions' and 'absolutely not' (Appendix). Internal consistency of the belief measure for this sample was .94. The *Doping behaviour *latent variable was defined by two self-reported measures of doping behaviour: current use of and past experience with performance enhancing substances.

The *Sport Orientation Questionnaire (SOQ) *is a multidimensional, sport specific measure of individual differences in sport achievement orientation. The questionnaire contains 25 items that uniquely relate to one of three independent factors: (a) competitiveness, (b) winning, and (c) goals. Competitiveness is defined as "the desire to enter and strive for success in sport competition" p.200 [50]. The desire to win in a sport situation is a sport specific measure and not related to general individual achievement orientation. Goal orientation reflects an orientation to personal standards, regardless of the situation. Of the total 25 items, the competitiveness subscale consists of 13 items, whereas the winning orientation and goal orientation subscales contain 6 items each. Respondents are asked to indicate how they usually feel about sport and competition on a five-point Likert scale that ranges from strongly agree to strongly disagree. The internal consistency coefficients for the three subscales are reported as follows: competitiveness subscale 0.94, win orientation subscale 0.86, and goal orientation subscale 0.80 [50]. In the present study, the observed internal consistencies of the SOQ were excellent: Competitiveness (α = .98), Win orientation (α = .93) and Goal orientation (α = .96). Reliability of the *Social Desirability *(SD) scale was less than desirable (*KR-21 *= .64), although *KR-21 *values are known to produce conservative estimates. Due to the nature of responses (Y/N where there is no correct or incorrect answer), using the more general but more conservative reliability measure *KR-21 *is the appropriate approach. Weak but statistically significant correlation was only found between SD and PEAS (*r *= -.220, *p *< .001).

## Results

Cases were subject to both univariate and multivariate screening. Measures of goal orientation, win orientation and competitiveness were negatively skewed whilst self reported doping behaviour measures were positively skewed. PEAS and SD scores were normally distributed (Table 1).

**Table 1:** Measurement level descriptive statistics and univariate and multivariate normality (N = 174)

Variable	min	max	mean	sd	skew	c.r. (skew)	kurtosis	c.r. (kurtosis)
Doping belief	.00	4.00	1.057	1.248	.822	4.425	-.331	-.892
Competitiveness	13.00	65.00	57.890	13.265	-2.362	-12.718	4.662	12.552
Win orientation	6.00	30.00	25.620	6.072	-1.795	-9.665	2.556	6.882
Goal orinetation	6.00	30.00	26.780	5.592	-2.382	-12.829	4.962	13.360
Doping attitude	19.00	79.00	42.138	12.190	.505	2.718	-.223	-.600
Past use of doping	.00	3.00	.910	.949	1.173	6.315	1.203	3.238
Current doping use	.00	3.00	.580	.629	1.164	6.270	2.700	7.271
Multivariate Mardia's coeff.							26.185	15.385

Observations farthest from the centroid (Mahalanobis distance) (Group number 1)

Due to the multivariate non-normality of the data, the overall model fit was also tested using the Bollen-Stine corrected *p*-value, which indicates the probability of the perfect fit of the proposed model to the population; thus it is interpreted the same way as the *p*-value belongs to the *chi-square *statistic. As recommended by Byrne [62] and Kline [69], 500 bootstrapped samples were used to generate parameter estimates, standard errors of parameter estimates, and significance tests for individual parameters.

### Descriptive statistics

First, descriptive statistics were calculated. Descriptive statistics on measurement level variables are provided in Table 1. (Due to the size of the item level matrix, it is not provided but is available upon request, as well as item level descriptive statistics.) Percentages of self-reported use and past use of performance enhancement drugs were congruent with the literature but due to the sensitivity of the questions, they were probably underreported. Notably but not surprisingly, past use of doping was admitted more than present use. Of the 199 athletes, 15 (7.5%) reported having personal experience with doping and an additional 9 (4.5%) claimed to have used substances classified as doping for medical reasons. The same figures for current use of performance enhancing substances were lower: 5 (2.5%) and 1 (0.5%), respectively.

### Item level analysis

Due to the difference in measurement scales, the first analysis was done at the item level. The model was recursive with *df *= 977. Standardized regression weights are displayed in Table 2.

**Table 2:** Item level parameter estimates and significance (at .05 level denoted by *)

			Std Regr. Weights estimate				Std Regr. Weights estimate
Doping Attitude	←	Competitiveness	-2.528	LegalAll	←	Doping Belief	0.911*
Doping Attitude	←	Goal orientation	1.603	D.Experience	←	Doping Behaviour	0.883*
Doping Attitude	←	Win orientation	0.935	Current use	←	Doping Behaviour	0.478*
Doping Attitude	←	Doping Belief	0.592*	SO24	←	Goal orientation	0.877*
Doping Behaviour	←	Competitiveness	2.045	SO20	←	Goal orientation	0.939*
Doping Behaviour	←	Win orientation	-0.364	SO16	←	Goal orientation	0.898*
Doping Behaviour	←	Goal orientation	-1.700	SO12	←	Goal orientation	0.886*
Doping Behaviour	←	Doping Attitude	- 0.076	SO8	←	Goal orientation	0.868*
Doping Behaviour	←	Doping Belief	- 0.312*	SO4	←	Goal orientation	0.914*
PEAS1	←	Doping Attitude	0.677*	SO2	←	Win orientation	0.917*
PEAS3	←	Doping Attitude	0.630*	SO6	←	Win orientation	0.804*
PEAS4	←	Doping Attitude	0.526*	SO10	←	Win orientation	0.903*
PEAS5	←	Doping Attitude	0.509*	SO14	←	Win orientation	0.570*
PEAS6	←	Doping Attitude	0.291*	SO18	←	Win orientation	0.866*
PEAS8	←	Doping Attitude	0.436*	SO22	←	Win orientation	0.884*
PEAS10	←	Doping Attitude	0.549*	SO25	←	Competitiveness	0.933*
PEAS11	←	Doping Attitude	0.471*	SO23	←	Competitiveness	0.903*
PEAS15	←	Doping Attitude	0.330*	SO21	←	Competitiveness	0.932*
PEAS17	←	Doping Attitude	0.402*	SO19	←	Competitiveness	0.803*
PEAS19	←	Doping Attitude	0.478*	SO17	←	Competitiveness	0.915*
PEAS21	←	Doping Attitude	0.373*	SO15	←	Competitiveness	0.903*
PEAS22	←	Doping Attitude	0.663*	SO13	←	Competitiveness	0.828*
PEAS23	←	Doping Attitude	0.297*	SO11	←	Competitiveness	0.886*
PEAS25	←	Doping Attitude	0.532*	SO9	←	Competitiveness	0.929*
PEAS28	←	Doping Attitude	0.212*	SO7	←	Competitiveness	0.952*
PEAS29	←	Doping Attitude	0.539*	SO5	←	Competitiveness	0.964*
LegalTop	←	Doping Belief	0.976*	SO3	←	Competitiveness	0.929*
				SO1	←	Competitiveness	0.909*

PEAS_i _= Performance Enhancement Attitude Scale items; SO_i _= Sport Orientation Questionnaire items; LegalTop = legalising doping for athletes at the top level of sport; LegalAll = legalising doping for all athletes; D.Experience = past use of doping' Current use = current use of doping.

Items defining sport orientation subscales had very high regression weight often close to 1.00. The correlations between sport orientation measures were very strong and positive; correlation coefficients for win orientation, goal orientation and competitiveness were above .93 for all pairs. Modification Indices suggested specifying relationships among items within and between the scales, which suggest multicollinearity and having standardized regression weights above 1.00 and *r*s > .85 indicate the same. Very high correlations among the items; and individual items and their factor are especially present in the Sport Orientation Questionnaire.

Overall, the item level model showed poor fit. Whilst some goodness of fit indices (e.g. *χ*^2 ^= 2091.9, *df *= 977, *p *= .880, *χ*^2^/*df *= 2.141 using the ML chi-square test as suggested by Marsh, Hau and Wen [70] met the conventional cut off criteria for goodness of fit indices (*TLI *= .855; *CFI *= .863) fell short of the more stringent criteria proposed by Hu and Bentler [71]. *SRMR *= .0797; *RMSEA(90CI) *= .081(.076, .086) were higher than the conventional standards of .08 and .06, respectively. Bootstrapped Bollen-Stine *p *value = .010 led to the same conclusion.

### Measurement level model

Summated scales may not have the multicollinearity problem caused by individual items being very similar. Thus, the same model of doping was tested with summed scale values. Moreover, using sum scores instead of individual item values helped to overcome the problem of categorical variables. Summed scores from psychological tests with items measured on at least a 5-point scale are generally treated as continuous variables [62].

The measurement level hypothesized model of doping is depicted in Figure 1. The hypothesized model of doping was recursive. Correlation coefficients among the measured variables are shown in Table 3. Goodness of fit statistics were statistically non-significant at the .01 level but the model should be rejected at the .05 level (*χ*^2 ^= 16.74, *df *= 7, *p *= .02, *χ*^2^/*df *= 2.39). However, the relative chi square was under the recommended 3:1 range ([69]) indicating acceptable fit. Other fit indices (*TLI *= .966; *CFI *= .989; *SRMR *= .096; *RMSEA(90CI) *= .090(.039, .146)) also demonstrated a good model fit, even if the more stringent criteria of Hu and Bentler [71] are applied. Hoelter's critical N values suggest that the model would have been accepted at the .05 significance level with 146 cases and the upper limit of *N *for the .01 significance level is 191. No Modification Index was above the customary cutoff value of 4.00. Because the data violated the normality assumption, bootstrapped chi-square values were also calculated and of the 500 random samples, the model in 488 bootstrap samples fit better and only 12 fit worse or failed. The Bollen-Stine *p *= .026 provided further reassurance about the model fit.

**Table 3:** Correlation (*r*) matrix of sport orientation and doping orientation^a^, N = 174

	1	2	3	4	5
1. Doping attitude (PEAS)	1.000				
2. Doping Belief^b^	.399	1.000			
3. SOQ-Competitiveness^c^	(-.128)	(-.050)	1.000		
4. SOQ-Win orientation	(-.029)	(-.052)	.904	1.000	
5. SOQ-Goal orientation	(-.135)	(-.082)	.965	.861	1.000

^a ^non-significant correlation (α = .05) coefficients are in parentheses

^b ^Kendall's tau

^c ^SOQ: Sport Orientation Questionnaire

Among the regression weights (Figure 1 and Table 4), the only significant relationship was between doping belief and attitude. Self reported doping behaviour had a significant relationship with doping belief, but not with attitude or sport orientation. Of the sport orientation measures, win orientation showed significant relationship with doping attitude. Correlation among the sport orientation measures (Table 5) were very high, all three being above .80 and naturally, significant. Variances, corresponding critical ratios and significance levels are displayed in Table 6.

**Table 4:** Measurement level standard regression rates, parameter estimates, standard errors (s.e.), critical values (c.r.) and significance (p)

Paths	Std regression weights	Estimate	s.e.	c.r.	p
Goal orientation → Doping attitude	.115	.236	.516	.458	.647
Win orientation → Doping attitude	.344	.685	.307	2.234	.025
Competitiveness → Doping attitude	-.436	-.397	.272	-1.460	.144
Doping belief → Doping attitude	.482	4.670	.636	7.345	.000
Doping attitude → Doping behaviour	.032	.001	.002	.331	.741
Doping belief → Doping behaviour	.324	.078	.038	2.080	.038
Goal orientation → Doping behaviour	-.238	-.012	.017	-.731	.465
Win orientation → Doping behaviour	.044	.002	.010	.226	.821
Competitiveness → Doping behaviour	.191	.004	.009	.501	.616
Doping behaviour → Current use	.479	.412	.173	2.389	.017
Doping behaviour → Past use (experience)	.880	2.426	1.015	2.389	.017

**Table 5:** Variances, covariances and correlations of winning, goal orientation and competitiveness measures

			Correlation estimate	Covariance Estimate	Standard error	Critival value	Sig. (p)
Win orientation	↔	Competitiveness	.904	72.370	8.207	8.819	< .001
Goal orientation	↔	Win orientation	.861	30.639	3.569	8.584	< .001
Goal orientation	↔	Competitiveness	.965	74.997	8.211	9.134	< .001

**Table 6:** Variances of winning, goal orientation and competitiveness measures and measurement errors

	Estimate	Standard error	Critical value	Sig. (p)
Goal orientation	34.519	3.711	9.301	< .001
Win orientation	36.661	3.942	9.301	< .001
Competitiveness	174.952	18.811	9.301	< .001
Doping belief	1.548	.166	9.301	< .001
Error (doping attitude)	108.289	11.643	9.301	< .001
Error (doping)	.079	.037	2.142	.032
Error (current doping use)	.302	.049	6.229	< .001
Error (past doping use)	.155	.213	.729	.466

Owing to the lack of previous empirical research, with the exception of sport orientation and self-reported use of doping, there was no other study for comparison. Furthermore, the data used for this model may warrant some caution. In general, covariance structure analysis, like other parametric statistical procedures, assumes that variables are being measured on a continuous (numerical) scale. As was discussed earlier, psychological scales that have at least 5 points are generally treated as continuous variables. The doping behaviour, however, was measured on a weaker scale with only 3 interval points (use, use for medical reasons, and no use).

The risk related to treating discrete variables as continuous variables for structural equation modeling has been reviewed and thoroughly discussed by Byrne [62]. The consequences of such violations lead to lower Pearson correlation coefficients when the two variables are not continuous, thus the models' regression weights might be conservative estimates. The situation is even worse, when there are less than five categories and/or the data are highly skewed. In relation to skewness, the worst scenario is when variable distributions are skewed in opposite directions. Although the present data were skewed, the variables 'belonging together' were skewed in the same direction. Athletes generally scored very high on sport orientation measures and below the mean/median on the doping related measures. On the other hand, normally distributed categorical variables have very little effect on the chi-square likelihood ratio of the model fit. Thus, as it is recommended, instead of relying on the goodness of fit test (chi-square), several fit indices were used to evaluate the hypothesized models. Finally, serious underestimation of factor loadings and factor correlations can occur when the number of categories is less than three. With category points between three and five, a modest underestimation should be expected. However, recent studies showed that serious problems are associated with dichotomous scales and methods relying on continuous data can be used with scales of four or more categories with no or little worry (p. 72 [62]). Keeping in mind that underestimation can occur when a variable is measured on a scale with less than four points; it is possible that the path between doping behaviour and its indicators would be stronger when measured differently. However, the regression weights associated with each were already statistically significant.

## Discussion

One of the most important features of the present study was the finding that sport orientation is not strongly related to doping behaviour, or to doping attitude. The only exception was win orientation, which showed a significant relationship with doping attitude. Thus the importance of winning may have influenced what athletes think about doping, but it does not necessarily manifest in their behaviour. From the path coefficients, it was clear that athletes' desire to win, to achieve their personal goals or their competitive nature is not necessarily related to their decision regarding use of prohibited performance enhancements. None of the measures, except expressed belief, had a significant path to behaviour. Apparently, athletes using prohibited means of performance enhancements do not have to be overly competitive or win-orientated. They do not have to endorse such pharmaceutical agents, or agree with the use of such substances in order to actually use them. These findings seem to be congruent with conclusions that emerged from previous qualitative studies [46] stating that doping is often viewed as a necessary means to an end. Many athletes claimed that they would prefer not to use drugs and would not do it if they were certain that the competition was drug-free. The paranoia about other competitors using performance enhancement is a reappearing theme in these papers. In addition, Anshel [72] noted that athletes often feel an external pressure to win, most often in the form of warning about exceptionally good opponents. Hence, using doping agents may be more of a rational, outcome optimizing behaviour than deviance. If this is the case, sport governing bodies may do well if in addition to placing a ban on certain performace enhancing substances and methods, they provide athletes with acceptable alternatives.

The small negative (but not significant) relationship between goal orientation and doping behaviour was a logical connection because among the three sport orientation measures, goal orientation reflects an orientation to personal standards, regardless of the situation. The other two measures, desire to win and competitiveness reflect a tendency to enter and strive for success in a sport situation. Using banned performance enhancements in most athletes' view was expected to be against their standards as sportsmen. However, at the same time doping is often viewed as a means to an end; a 'tool' that is bad but necessary to ensure success in competition. Therefore, a positive relationship was expected. Of the two measures studied, competitiveness had a small but insignificant, positive path to doping behaviour, whilst winning practically showed no relationship at all. On the other hand, the only statistically significant relationship with sport orientation measures and other factors was between win orientation and doping attitude. Sport orientation and attitude appear to be similar constructs and distinctly different from behaviour. Athletes may *think *that doping is needed or not needed for winning but when it comes to actual *behaviour*, it might be influenced by other factors more than attitude or orientation.

This is not to say that personality, attitude, values should be discarded in order to make room for other factors. As probably no two individuals would react identically to the same combination of environmental factors, it is fair to assume that contextual contingencies are mediated through the combination of individual factors. Adherence to norms is a particularly difficult question. Decisions regarding doping use are influenced by at least two possibly competing norms: 1) the general social norms, such as fair play, condemnation of cheating and 2) the special norms held by the athletes' immediate subcultures as suggested by English [48] particularly to competitive sport. When respondents completed the survey, they might feel compelled to consider the general social norms and offer a picture of a fair playing athlete. Athletes might answer in a particular way so they were seen as highly motivated, goal oriented individuals who understandably placed great importance on winning and achieving in a competitive situation (as the highly skewed sport orientation measures suggest) but despised unaccepted means of performance enhancement (again, mean score was rather low on doping attitude). The low correlation between the sport orientation and doping attitude measures and the Social Desirability scale gave some reassurance that the data were not contaminated badly by response bias but its effect warrants further investigation.

The non-significant path between doping attitude and behaviour was surprising. Instead of the practically zero regression weight, a small positive but significant relationship was expected. Although the *Theory of Reasoned Action *[73] and *Theory of Planned Behaviour *[42] suggest that beliefs form attitude before their effect on behaviour, results from this research showed a fairly strong and significant direct path from beliefs to behaviour. Mediating beliefs through attitude would only slightly increase the regression weight between doping attitude and behaviour but remained non-significant (*β *=.14, *p *= .446). One possible explanation for the strong path between belief and behaviour is justification. Those athletes who use doping or wish to use such performance enhancements would prefer to do so without social stigmatization. Such a view is in keeping with previous research where athletes expressed their view of doping as a necessary means to a desired end and whilst they acknowledge rule breaking behaviour, they do not consider themselves cheaters or more cheating than any other athlete [74].

Follow-up research efforts should be directed toward finding additional components that may contribute to the doping model, such as drug attitude, morality, anxiety over performance, health concerns, and deterrent factors. Data collected via self-reports always pose limitations to the study owing to the undesirable but unavoidable effect of response bias (typically resulting in the under-reporting of socially undesirable behaviour). Modelling behavioural intentions in hypothetical situations instead of self-reported actual behaviour may help to reduce the effects of strategic responding. The appropriateness of testing via hypothetical situations is discussed in Strelan and Boeckmann's perceptual deterrence model paper [28]. Alternatively, experimenting with implicit measures, as oppose to explicit self-declaration, may also provide a useful approach to doping behaviour reseach.

The relationship between doping belief and behaviour was significant, suggesting that investigating and quantifying belief might be a more fruitful approach in the future. Individual attitudes or actions, however, cannot be understood without taking the environmental context into consideration. The environmental effect is used in a broader sense, as it should include the culture (i.e. country) and subculture (i.e. sport), societal norms (i.e. values which society holds about sport and in general) but also the influence of other people (i.e. peers, coaches, family), alternative choices, and consequences of acting or not acting in a certain way.

Whilst the relationship between behavioural intention and behaviour is well established, moderating factors are less commonly used. Godin, Gagnë and Sheeran [75] provided empirical evidence from meta-analysis of eight published health-related behavioural intention research for the importance of including moderating variables, such as perceived behavioural control (a combination of perceived difficulty and perceived control) and perceived power. Including these elements will further enhance the doping model.

Doping in varied cultures is perhaps viewed differently by the individual, his/her immediate social circle and broader society. The view of doping can even differ from one subgroup to another *within *the boundaries of sport. Therefore, testing for measurement invariance across groups (model's applicability to other groups, such as females, athletes from other nations or from different levels of sport involvement) should also be an important avenue to pursue in future research.

## Conclusion

The SEM analyses provided empirical evidence that sport orientation and doping behaviour is not directly related. The understanding we can gain from research aiming at individual characteristics is limited if the context was ignored. Sport governing bodies and anti-doping organisations need to recognise that using performance enhancements (both acceptable and prohibited methods) may be more of a rational, outcome optimizing behaviour than deviance.

There is an important lesson to be learned from mainstream management. Similar lines of research have been conducted in leadership when the first model in search of 'good leaders' exclusively focused on the leader and his or her personality and only decades later reached the point when complexity of the phenomena and contextual contingencies were acknowledged and taken into account. At this point, the factors that make the difference between using and not using banned performance enhancements are not well understood. Focusing solely on athletes' personalities and treating doping as a deviant behaviour of the few is misleading. No doubt, attitude is an important construct in social psychology and plays a significant role in the doping issue as well. However, one must be careful not to place too much emphasis on attitudes to the exclusion of other, perhaps equally important factors. Athletes' sport orientation appeared to be unrelated to doping behaviour. Doping specific attitudes, but more so beliefs, are an integral part of doping behaviour, but it cannot be said whether they are reasons or consequences of other, related attitudes, dispositions and experiences. Attitudes and actions are intimately intertwined and such unison is subject to constantly competing forces to maintain the inner equilibrium (as a natural human desire for stability) *and *to change in accordance with new experiences, information and situation. Instead of focusing solely on attitudes, a better understanding of doping should be developed. Perhaps then, alternative actions can be identified, explored, and offered for athletes who wish to constantly improve their performance up to or above the level of competitors.

The results of this research have both theoretical and practical implications. At the theoretical level, the findings of this paper are a step toward a comprehensive doping model. This paper highlights the need for the inclusion of other influencing factors and makes suggestions for future model testing. At the practical level, understanding the driving forces behind doping and how athletes wish to deal with these factors must be at the centre of informed deterrence policies. Athletes are, by nature, highly motivated and achievement oriented individuals and have grown to appreciate methods for performance enhancement (training, nutrition, physiotherapy, equipment, etc.). The distinction between acceptable and prohibited methods must be made clear and convincing. To be effective, authorities must be able to i) justify the doping ban in general, ii) use evidence-based selection of substances and methods included into the prohibited list, iii) use the same criteria for all substances and methods, and iv) communicate such decisions to all stakeholders.

Suggesting anti-doping education and perhaps changes in attitudes to doping is a rather futile approach if the other influencing factors are kept constant. A value-based deterrence requires changes at all levels and in all stakeholders. Large scale research aiming to understanding the driving forces behind doping behaviour and gaining knowledge of effective deterrent factors is much needed and should be extended beyond the athlete population to include coaches, managers and officials. Sport governing bodies and anti-doping organisations are in the unique position to endorse and foster such research. International and national anti-doping organisations should make targeted funding opportunities for doping-related research aiming at increasing knowledge regarding both the doping behaviour and alternative acceptable means of performance enhancement. Constant improvement of performance is, after all, the core characteristic of competitive sport.

## Competing interests

The author(s) declare that they have no competing interests.

## Appendix

### Performance Enhancement Attitude Scale (PEAS)[53]

1 = Strongly disagree, 2 = Disagree, 3 = Slightly disagree, 4 = Slightly agree, 5 = Agree, 6 = Strongly agree

1. Doping is necessary to be competitive.

2. Doping is not cheating since everyone does it.

3. Athletes often lose time due to injuries and drugs can help to make up the lost time.

4. Only the quality of performance should matter, not the way athletes achieve it.

5. Athletes in my sport are pressured to take performance-enhancing drugs.

6. Athletes, who take recreational drugs, use them because they help them in sport situations.

7. Athletes should not feel guilty about breaking the rules and taking performance-enhancing drugs.

8. The risks related to doping are exaggerated.

9. Athletes have no alternative career choices, but sport.

10. Recreational drugs give the motivation to train and compete at the highest level.

11. Doping is an unavoidable part of the competitive sport.

12. Recreational drugs help to overcome boredom during training.

13. There is no difference between drugs, fibreglass poles, and speedy swimsuits that are all used to enhance performance.

14. Media should talk less about doping.

15. The media blows the doping issue out of proportion.

16. Health problems related to rigorous training and injuries are just as bad as from doping.

17. Legalising performance enhancements would be beneficial for sports.

### Doping Use Belief (DUB)

Do you believe that performance-enhancing drugs/methods should be allowed for top level athletes?

Yes, without restrictions (2), Yes, but with restrictions (1), Absolutely not (0)

Do you believe that performance-enhancing drugs/methods should be allowed for all athletes?

Yes, without restrictions (2), Yes, but with restrictions (1), Absolutely not (0)

Have you ever had personal experience with banned performance-enhancing drugs and/or methods?

Yes (3), Yes, but only for treating a medical condition (2), No (0), I do not wish to answer (1)

Do you currently use banned performance-enhancing drugs?

Yes (3), Yes, but only for treating a medical condition (2), No (0), I do not wish to answer (1)
